# Does information form matter when giving tailored risk information to patients in clinical settings? A review of patients’ preferences and responses

**DOI:** 10.2147/PPA.S125613

**Published:** 2017-03-01

**Authors:** Rebecca Harris, Claire Noble, Victoria Lowers

**Affiliations:** Institute of Psychology, Health and Society, University of Liverpool, Liverpool, UK

**Keywords:** risk, patient communication, personalisation, information, behavior change, health education

## Abstract

Neoliberal emphasis on “responsibility” has colonized many aspects of public life, including how health care is provided. Clinical risk assessment of patients based on a range of data concerned with lifestyle, behavior, and health status has assumed a growing importance in many health systems. It is a mechanism whereby responsibility for self (preventive) care can be shifted to patients, provided that risk assessment data is communicated to patients in a way which is engaging and motivates change. This study aimed to look at whether the form in which tailored risk information was presented in a clinical setting (for example, using photographs, online data, diagrams etc.), was associated with differences in patients’ responses and preferences to the material presented. We undertook a systematic review using electronic searching of nine databases, along with handsearching specialist journals and backward and forward citation searching. We identified eleven studies (eight with a randomized controlled trial design). Seven studies involved the use of computerized health risk assessments in primary care. Beneficial effects were relatively modest, even in studies merely aiming to enhance patient–clinician communication or to modify patients’ risk perceptions. In our paper, we discuss the apparent importance of the accompanying discourse between patient and clinician, which appears to be necessary in order to impart meaning to information on “risk,” irrespective of whether the material is personalized, or even presented in a vivid way. Thus, while expanding computer technologies might be able to generate a highly personalized account of patients’ risk in a time efficient way, the need for face-to-face interactions to impart meaning to the data means that these new technologies cannot fully address the resource issues attendant with this type of approach.

## Introduction

Risk communication is something that most clinicians do every day.^1^ This is because, first, patients’ risk perception (belief about the likelihood of personal harm from a behavior), and how this balances with benefits, lies at the heart of helping patients make informed choices between treatment options and, second, because self-care and self-management behavior is underpinned by how patients perceive threats to their health.^2^,^3^ Risk communication is also the concern of public health practitioners, where it is seen as crucial to the prevention and cooperative management of health risks, and “at least equally essential to outbreak control as epidemiological training and laboratory analysis”.^4^ Literature on health risk communication is, therefore, understandably prolific – embracing a range of disciplines and theories that explore the complexities of how individuals are influenced by such information.^4^

There is a general consensus that tailoring of information is beneficial,^5^–^7^ and so we set aside “mass” programs concerned with risk communication and focus here on communicating individualized information. Individualized health communication can range from personalized generic communication (for example using someone’s name to personalize the message), to targeted communication (composing the message with a particular group or segment of the population in mind – an approach that is the basis of many public health education and social marketing campaigns), through to truly personalized communication that provides information based on characteristics unique to a person (as in brief counseling interventions, for example). These latter approaches involve tailoring based on characteristics beyond broad demographic categories such as age or gender, and therefore depend on some sort of individual assessment; although with the advent of computer-based tailoring, their population reach can still be wide.^8^,^9^

A common aim of tailoring used in health education messages is to increase attention and therefore message comprehension – both cognitive preconditions for the processing of information, which lead to a change in behavior.^6^ It is also thought that tailoring works by way of peripheral or emotional processing; for example, “the sender understands me”; which enhances source credibility and the following of recommendations with little critical analysis.^6^ Some even argue that patients’ assessment of risk is primarily determined not by facts but by emotions,^10^ for the more risk information evokes an emotional response, the greater the perceived chance of the threat occurring.^11^

Studies show that visual displays enhance people’s understanding of risk, particularly holding attention when they are given in a vivid way;^12^,^13^ and emotional responses to information portrayed say in pictures or videos influence whether people increase or decrease certain health behaviors.^12^ So, although much previous attention has been focused on the way risk messages are framed and presented (comparing gain-framed with lost-framed messages and various numerical and graphical formats),^1^,^14^ the actual form in which the risk information is presented (verbal, written leaflet with or without diagrams, video, computer, photograph) is an important additional feature that may influence people’s engagement and responses to the material. With current expansion in possibilities of tailored risk communication by means of intelligent interactive systems,^15^ it is important to consider both patient preferences and their responses to risk information when presented in different forms. Our aim was therefore to undertake a systematic review of patients’ preferences and responses to personally tailored information given in different forms, limiting this to clinical settings (“patient communication”), although the work may inform wider public health education efforts too. After presenting the results of the review, we go on to discuss what this means in modern times where computer and mobile phone capabilities make it possible to issue a wealth of feedback on lifestyle and clinical information to patients against a background where health policies increasingly advocate efficiencies of care delivery and patients’ responsibility for their own health.

## Methods

Literature searching was limited to all types of study design, including qualitative work and protocols, concerned with adult patients receiving tailored risk information as part of their care in clinical settings. Intervention studies were only included where the study involved comparing delivery of tailored risk information in one form with either usual care, verbal risk messages, or with a different form of risk information so that a comparison regarding differing information forms could be made (Table 1 shows full inclusion and exclusion criteria). Since studies show that lay concepts of “risk” tend to be more aligned with a dichotomous model of risk presentation (“I am a likely/unlikely candidate for illness”), than a model involving graduations along a probability spectrum (“I am at a 30% higher risk of being ill than someone else of my age”),^16^ we included studies involving giving tailored information about individuals’ levels of health with reference to likely negative consequences as well as those involving “risk” terminology and health outcome probabilities.

We adopted an iterative search strategy that involved electronic literature searching of nine databases (including gray literature and dissertation databases) and handsearching eight specialist journals (Supplementary material Table S1). To strike a balance between literature search sensitivity (finding all articles in the topic area) and precision (finding only relevant articles), we initially developed electronic search terms using Automatic Term Recognition software (TerMine), applying this to 35 papers previously retrieved through pilot searches undertaken in Google Scholar.^17^ We then broadened out the search strategy with general topic search terms (eg, health education) as is customary to systematic review methods.^18^ We also used forward and backward citation searches, that is, reviewing references cited in articles identified earlier in the review process and searching for publications which cited papers that met study inclusion criteria. Quality assessment of included randomized controlled trials (RCTs) was undertaken using Cochrane risk of bias methodology.^19^

## Results

**Figure 1:**
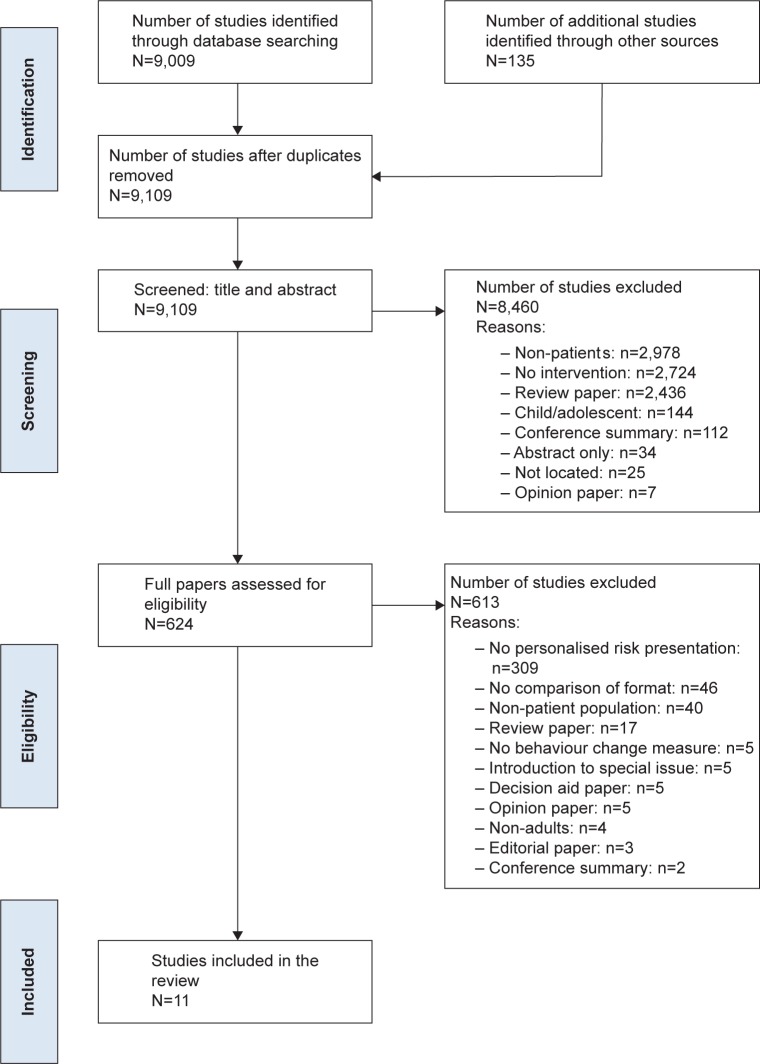
PRISMA diagram. **Abbreviation:** PRISMA, Preferred Reporting Items for Systematic Reviews.

Electronic and handsearching identified 10,682 papers, of which 1,673 were duplicates. A further 100 papers were identified through backward and forward citation chasing. Screening by two independent reviewers identified 624 relevant papers. Full-paper screening by two reviewers left eleven included papers,^20^–^30^ (Figure 1). The most common reason for paper exclusion (309) was because the risk information presented was not fully personalized as set out in our inclusion criteria (requiring a patient assessment prior to receiving the information, Table 1). In the majority of these excluded papers, risk information was formulated using broad population characteristics such as age. Another 51 papers were excluded because they involved considering only one form of presenting information to patients, rather than a comparison between two different forms or comparing a certain form of information (eg, photographs) with verbal information or usual care. Full reasons for exclusion are given in Figure 1.

Details of included papers indicate that this is a relatively new research area (Table 2). Eight of the eleven papers were published in the last 5 years. No studies were found that made comparisons between different information forms, with most included studies comparing particular forms of communicating risk information with usual care. Heterogeneity in study design and outcomes of included studies meant that a meta-analysis was not undertaken. Where data from reviews are insufficient to merit pooling of included studies because of the very wide range of interventions covered, a “narrative synthesis” is recommended.^31^ Narrative synthesis involves summarizing the main features of different studies and important characteristics (such as similarities and differences between studies) and identifying patterns of results in the data.^31^

### Summary of included studies

Five studies concerned cardiovascular risk information,^23^,^24^,^26^–^28^ one concerned asthma risk information,^29^ and the rest covered broader “healthy life check” information. Three studies involved information for Type 2 diabetes patients.^24^,^28^,^30^ Although eight studies used an RCT design, two were feasibility studies^23^,^26^ and two were pilot RCTs.^25^,^27^ Of the three remaining publications, one was an intervention description,^30^ one a protocol,^27^ and the other an uncontrolled prospective study.^28^ Quality assessment of included RCTs indicates that some of the RCTs had a low risk of bias in many domains, apart from intervention and outcome assessment blinding (Table 3).

### Computer generated individualized written feedback on health risk

Seven articles concerned personalized risk information presented on computer.^20^–^23^,^25^,^29^,^30^ Developments in information technology have made it possible to combine health behavior change theory, communication theory, social marketing principles, and computer-based programs and algorithms to produce personally relevant health messages for individuals. Information from participants’ survey data can be assembled to generate customized messages, to the extent that it includes elements such as an individuals’ health literacy, locus of control, internet experience, attitude to self-care, decision preferences, and current health knowledge.^30^ Computer technology allows incorporation of several hundred text files, graphics, and photographs which can potentially correspond with each survey question selected for tailoring and its possible response option combinations.^32^ By personalizing messages and the language in the interactive dialogue (for example, contextualizing according to the user’s viewpoint eg, “as you said before …”), attention and impact is thought to be increased.

Most of the randomized controlled trials within our included studies involved computer-generated health risk appraisals (HRA), although results were generally disappointing. An RCT of a web-based intervention delivering personalized cardiovascular risk information to patients was found to be ineffective, with no significant differences in health outcomes or behavior between intervention and control groups after 3 months.^23^ Even a study of computerized HRA where the outcome of interest was set relatively modestly at changes in risk perception found that adjustments in optimistic and pessimistic bias only occurred in some of the disease domains studied^22^ (Table 2).

Two included studies reported randomized controlled trials of computerized HRAs administered in a general medical practice setting.^20^,^21^ Both involved older adults. The earliest of these integrated computerized HRAs into practice-based information technology systems and generated individualized feedback to both patients and general practitioners who had been trained on current care and behavior recommendations relating to the risk domains covered. It was, however, left to the discretion of doctors and patients as to how any issues identified were addressed in consultations, if at all.^21^ Results were relatively disappointing, with minimal improvement in patients’ health behavior or uptake of preventive care across the domains studied^21^ (Table 2). Intervention group participants reported slightly higher pneumococcal vaccination uptake (odds ratio [OR]: 1.7, confidence interval [CI]: 1.4–2.1) and some improvement in physical activity levels compared with controls (OR: 2.0, CI: 1.6–2.6). However, no significant differences were observed for any other of the 14 categories of health behavior or types of preventive health service use at the 12 month follow-up.^21^

A later study, this time undertaken in medical practices in Hamburg, Germany, offered additional message reinforcement as well as the HRA information for patients and practitioners (again with a training of the general practitioners involved).^20^ Overall, results were slightly better (Table 2). While there were still no differences between intervention and controls in mortality, hospital admissions, and the frequency of visits to a doctor, there were small but statistically significant shifts in self-reported health behaviors.^20^ After 1 year, the proportion of 9 types of preventive service use (such as dental check-ups) was an average of 75% in the intervention group and 68% in controls (OR: 6.1, CI: 4.3–7.9).^20^ Likewise, out of six possible health behaviors (such as three or more moderate to strenuous physical activities per week), 64% of these behaviors were reported by the intervention group, versus 60% in the controls (OR: 3.7, CI: 2.0–5.4).^20^ Of the 804 participants in the HRA intervention group, 503 opted to take up some group session reinforcement, 77 opted for home visit reinforcement, and 224 did not take up the reinforcement offer. This allowed for a subgroup analysis to explore the efficacy of the reinforcement component within this complex intervention. Findings indicate that a reinforcement component is needed if the intervention is to be effective. The “difference” in reported preventive service use between intervention and controls was 7.1% (CI: 5.2%–9.0%; *P*<0.001) for those receiving the full HRA intervention, including some kind of reinforcement, but only 2.0% (CI: −2.2 to 6.3, *P*>0.1) where intervention participants received the HRA only.^20^ The same pattern was seen in other self-reported health behavior outcomes.^20^

Although authors suggest that computerized HRAs in clinical settings are best used to complement face-to-face consultations with clinicians, making them “more efficient and satisfying for both sides” by “increasing patients knowledge and power to enable them to be active partners in their care”,^30^ an RCT using computer-generated risk information on tablet PCs just prior to a doctor’s appointment does not support this.^25^ Little increase in both patients’ and doctors’ reports of discussion on various health topics for patients with prior access to their HRA was found.^25^ Harari et al^21^ also reported no HRA effect on patients’ self-efficacy related to patient/doctor interactions (Table 2). In summary, therefore, several studies come to the same conclusion: that although computerization makes tailoring of risk information possible, and enables simple and visual representation of complex risk information, additional input is needed to interpret and discuss the feedback – in other words, some sort of face-to-face component to HRA interventions appears to be needed if beneficial effects are to be seen.^20^–^23^

### Risk information presented by way of diagrams, charts, and photographs

These small or non-significant findings are not limited to risk information presented on computers. Studies in the clinical setting presenting risk information by way of population diagrams,^24^,^28^ colored charts,^26^ or photographs^27^ come to similar conclusions – that risk information presented in this way alone is insufficient to prompt patients to adopt healthier lifestyles or to enhance clinical communication (Table 2). The only effect found was a short-term increase in risk perception.^24^,^27^ Welschen et al^24^ conclude that risk communication is insufficient on its own, but should be a first stage in a more complex lifestyle intervention.

The RCT by Shahab et al^27^ using ultrasound scans showing the extent of blockage in carotid arteries allows some insight into the processes involved. They theorized that visual imagery such as scans of partially blocked carotid arteries span the conscious–unconscious continuum more readily than language, with the result that patients experience less filtering out of the information by the “conscious critical apparatus”, which usually serves to disengage the individual from beliefs which derogate the threat message. Their study collected behavior mediator variables based on the Extended Parallel Process model and was able to offer an explanation as to why some individuals were able to ignore the threat message even when it was presented in such a vivid way. Results showed that positive responses to the threat message presented were dependent on individuals having high self-efficacy beliefs (feeling able to make positive changes in the necessary behavior).^27^ A more recent study by Saver et al^28^ supports the hypothesis that individuals are able to distance themselves from computer-generated risk information, even when it is presented in an personally tailored way. Participants professed that “the computer model is wrong about me … I know my health better … than some statistics”. Almost 80% reported that they felt the data did not apply to them personally. Instead, 75% described “knowing myself” as an important way they understood their risks “because I know myself better than I think some statistics show ….” Embodiment of risk was described, although interestingly, the doctor was identified as someone who was the next best placed person to make risk judgments: “…. that’s why I go by my body experiences, besides the doctor, you are the one who knows how your body functions”.^28^

## Discussion

As is the case in all systematic reviews, despite carefully constructing electronic search strategies, some literature may have been missed if articles were poorly indexed. We recognize this as a possible limitation of the review. Systematic review search term filters are usually determined in a trade-off between sensitivity (ability to detect all possible publications on the topic, knowing that this will throw up a lot of papers not meeting inclusion criteria) and precision (ability to deliver a search identifying a high proportion of relevant papers).^33^ We attempted to balance these two considerations by undertaking text mining of sample papers, and then subsequently broadening the search to increase sensitivity, supplementing this with handsearching of specialist journals. However, it is possible that by using text mining to design a precise search, we may have limited its sensitivity somewhat, and so some relevant publications were missed.

Nevertheless, it is striking how little literature there is on how tailored risk information is received by patients in clinical settings, bearing in mind the emphasis on personal responsibility for health and providing personal health and lifestyle risk factor advice to patients which is the basis of current health policy in many countries.^34^ For example, in both medical and dental care in the UK, growing attention is paid to collecting a range of “life check” information using personal health and lifestyle risk assessment tools with the intention that this is linked to personalized advice to patients.^34^,^35^ This is in contrast to a wealth of studies contrasting whether people’s risk perception is best informed using various different types of numerical and diagrammatical representations.^14^ The expansion of technology that allows extensive personalization of risk information makes translation into the clinical setting tempting. Certainly, computer technology which allows a range of information to be incorporated into patients’ assessments on the face of it appears to offer some assistance to clinicians. However, our study indicates these approaches may be insufficiently meaningful for patients, to make this worthwhile on their own.

Results remind us that the very notion of “risk” itself differs substantially when approached from different standpoints. Scientific medicine defines “risk” in terms of an objective reality that can be measured, controlled, and managed.^36^ Although this approach tends to dominate thinking in this area of health care, and leads on to approaches which quantify risk, for example, with elaborate computer modeling of lifestyle data, our results indicate these may lack sufficient meaning for patients. In other words “risk” is something of a “trans-scientific” topic in that issues can be raised but not completely answered by science.^37^

Lindell et al^38^ identify that important differences exist when communicating risk information to individuals (in clinical settings) as opposed to populations. Science-based notions of risk which are based on mathematically expressed probabilities are only meaningful at the level of a population. Although this type of data represents objective, anonymized knowledge, at the level of individuals, the information becomes potentially emotionally charged and anxiety inducing.^38^ Lindell et al^38^ also observe that when talking to individuals about “risk”, it becomes concretized, almost “reified”, as if it was something “carried” by the patient in her own body – a conclusion which resonates with the qualitative data reported in Saver et al’s study.^28^

And so it is up to clinicians to “recontextualize” the information to make it meaningful at a truly personal level.^38^ Often data involving percentages are recast into an “all or nothing” scenario (“Will I get sick or not?”).^16^ And so we observe that clinicians naturally simplify risk information when talking to patients, to a relatively dichotomous model through the use of verbal qualifiers (“Your risk is high” or “This is not good for your health”).^39^ Misselbrook and Armstrong agree that when talking to individuals rather than populations, a high/low risk model is a better fit because it “provides the patient with a map to enable them to function and cope in an uncertain world”.^40^

A common theme across our included studies, which were limited to those undertaken in a clinical setting, is that “discourse” (in some sort of face-to-face interaction) is a necessary way in which meaning is imparted to risk information, making it possible to move from scientifically based risk representations relevant at a population level to notions of risk relevant to individuals. Our results indicate that this is still necessary with scientific data, even where this has been “personally tailored” to individuals. Faisal et al^41^ terms the process as “internalization of externalized data” (externalized data such as visual representations of data on computer-supported tools) and argue that “sense-making” is a necessary process of finding meaning from information. So, while risk information may be helpful in assisting people to perceive and make sense of their health status and medical condition, the process of sense-making concerns not just the data, but their own life experiences.^42^ The study by Dapp et al^20^ is particularly interesting because discourse on HRA data took place in groups or at home, and not in the medical practice with a doctor. These discursive practices help to define “who and what is normal, standard, and acceptable”.^43^ They help to challenge what was once “taken for granted”. It is after destabilizing current meaning that the information forms a basis for change.

## Conclusion

Although presenting personalized information on health risk to patients is increasingly expected as part of a general health policy approach that emphasizes patients’ contribution for their health by adhering to health education advice, our review reveals that relatively little empirical work has been done that compares the relative impact of communicating information on risk to patients using different forms. Most work has been done in the growing field of presenting computerized health risk appraisals to patients. Findings suggest, however, that the impact of this information format is limited because there remains a need for discourse between patient and clinician (or even between patients) in order to impart personal meaning to the information sufficient to prompt a change in behavior. More work is needed to explore this further.

## Supplementary material

**Table S1:** Electronic databases and journals searched

MEDLINE (Ovid MEDLINE and MEDLINE in process and other nonindexed citations)
Web of Science: Social Sciences Citation Index
Web of Science: Conference Proceedings Citation Index – Social Science and Humanities
PsycINFO
PsycArticle
Communication and Mass Media complete
Proquest Dissertations and Theses
Cochrane Library Cochrane Reviews (reviews and protocols)
Open Grey
Health Informatics Journal
Patient Preference and Adherence
Patient Education and Counselling
Health Communication
Journal of the American Medical Informatics Association
Preventive Medicine
Journal of Health Communication
BMC Medical Informatics and Decision Making

## Figures and Tables

**Table 1:** Inclusion and exclusion criteria

**Inclusion:**	
1.	Personalized (tailored) information given to patients, which is reliant on a preassessment of the patient rather than information targeted according to population characteristics such as age and gender
2.	Studies concerned with information aimed at increasing patients’ perception of health risk. These include studies involving tailored information about an individual’s level of health with reference to likely negative consequences, as well as those involving “risk” terminology or health outcome probabilities
3.	Studies reporting delivery of information in a certain form (eg, written, video, online, photograph) versus no intervention/usual care controls, or comparing information in different forms. In the control group, “usual care” information may or may not be tailored. Studies involving multicomponent interventions that had a control group component such as motivational interviewing, or education which was also part of the intervention group were included
4.	Outcome measures including one or more behavior mediators including risk perception, health behavior, health outcomes
5.	Adults aged 18 years +
6.	Patients receiving information as part of their care
7.	Any health system
8.	English language only
9.	Date: 1980 to present
10.	All types of study design including qualitative studies and protocols
**Exclusion:**	
1.	Studies concerned with giving information in a verbal form compared to a control
2.	Outcomes concerned with decision-making in relation to treatment options only

**Table 2:** Included papers: study design and main findings

Study	Participants	Intervention	Control	Follow-up	Outcome measures	Results summary
**Dapp et al**^20^ **(2011)** RCT Patients randomized by computer	Non-disabled Aged 60 years +21 medical practices Hamburg	N=878 (14 practices) **Written risk reports** Multiple risk factor Computer-generated HRA individualized written reports + personal reinforcement (choice of group session/home visit), + physician training	N=1,702 (14 practices) **Usual care** (with physician training and checklists with preventive recommendations) N=746 An additional 7 concurrent “comparison” practices with untrained GPs	1 year	**Behavior** 10 preventive care use behaviors (PCUB) eg, dental check-up 6 preventive health behaviors (PHB) eg, consumption of fruit or fiber **Health Outcomes** 5 measures eg, hospital admissions	**Adherence** ↑ in PCUB (OR: 1.7, CI: 1.4–2.1) and ↑ PHB (OR: 2.0, CI: 1.6–2.6) but subgroup analyses suggest a favorable effect only with personal reinforcement NS health outcomes **Preferences** Majority selected group rather than home visit reinforcement “Group reinforcement is promising”
**Harari et al**^21^ (**2008**) RCT Patients randomized by computer	Aged 65 years +4 general practices UK (26 GPs)	N=940 patients (18 GPs) **Written risk reports** Computer-generated HRA individualized written reports + letter encouraging discussion with doctor or PN + information on eg, exercise schemes + GP/PN training + GP summary report	N=1,066 **Usual care** (18 GPs) Concurrent comparison group (1 practice, 8 GPs)	1 year	**Behavior** 10 PCUB 4 PHB **Health outcomes** No. of hospital admissions No. of GP visits **Communication** Patient reported self-efficacy of patient/physician interaction	**Adherence** ↑ in 1 PCUB (OR: 1.2, CI: 1.01–1.5) and ↑1 PHB (OR: 1.4, CI: 1.0–2.0) NS health outcomes or patient self-efficacy Lower than expected effect attributed to lack of face-to-face reinforcement
**Kreuter and Strecher**^22^ **(1995)** RCT Randomization unreported	1,317 adult patients aged 18–75 years from 8 US medical practices	N= not reported **Graphical and numerical** presentation of patients 10-year mortality risk Group 1: HRA feedback Group 2: HRA feedback plus behavior change information Results combined groups 1 and 2 and only given for participants recalling the intervention	N= not reported Usual care	6 months	**Risk perception of mortality** Heart attack, stroke, cancer motorcycle accident Results reported for each mortality risk as perceived optimistic risk perception (unrealistically optimistic) and pessimistic risk perception (worried well)	↓ optimistic bias for risk perception of stroke mortality only (OR: 1.27, CI: 1.02–1.60) ie, intervention groups were 27% more likely to have ↑ risk perception at follow up ↓ pessimistic bias for cancer risk perception only (OR: 1.36, CI: 1.07–1.73) ie, intervention groups 36% more likely to ↓ risk perception at follow-up
**Zullig et al**^23^ **(2014)** Feasibility study Block randomization	US patients with CVD + a modifiable risk factor Mean age 65 years	N=96 **Web-based intervention** Given individual CVD risk face-to-face + link to self-directed online modules to adjust scores in areas where willing to change behavior	N=49 **Usual care** with general health education information	3 months	**Behavior** Medication adherence **Health outcomes** 10-year CVD risk score BMI, smoking Blood pressure	NS Web interventions may be ineffective without guidance and accountability from clinician interactions
**Welschen et al**^24^ **(2012)** RCT Patients randomized by computer	Referred T2D patients Netherlands	N=131 **Verbal** + **pictorial** Nurse gave a figure (%) for relative risk of CVD + visual risk card + population diagram + gather patient response through open questions + patient asked to “think aloud” explaining risk to themselves	N=130 **Usual care**	12 weeks	**Risk perception** Difference in actual and perceived CVD risk Anxiety and worry about CVD risk **Behavior** 6 attitudes and ICB diet, smoking and exercise **Communication** Communication satisfaction	Risk perception ↑ (β between group difference: 0.48, CI: 0.02–0.95) after 2 weeks, but not at 12 weeks (β between group difference: −0.03, CI: −0.43 to 0.37) NS risk anxiety/worry NS ICB There is no evidence that risk communication, besides an improved risk perception, will motivate patients to adopt a healthier lifestyle
**Hess et al**^25^ **(2014)** Pilot RCT Cluster randomized by doctor	Attending single US general practice Mean age 29 years	N=51 (16 doctors) **Computer-generated immediate feedback** of risk: tobacco use, physical activity, HRQoL before clinical appointment to prompt initiation of discussion	N=48 (14 doctors) **Usual care** (completing health questionnaire without feedback)	At the end of the visit	**Communication** PID reported by patient and doctor Patients reported to find the discussion useful (Unit of analysis = patients)	NS patient initiation of health-related discussion but ↑ doctor reports of PID on physical HRQoL only for patients with low physical HRQoL (OR: 4.6, CI: 1.3–16.3) **Preference**: NS patient perceived discussion to be useful
**Neuner-Jehle et al**^26^ **(2013)** Feasibility RCT Cluster randomized by doctor	Swiss general practice Median age 47 years Total 27 GPs 114 patients	**Verbal** + **numbers** + **pictorial risk message** GPs using “quit smoking tool” + individualized CVD risk calculation training presented in numbers and colored charts + training + guidelines including motivational interviewing	**Verbal** GPs using a “quit smoking tool” + training + guidelines including motivational interviewing	Not reported	**Behavior**: Before and after motivation using a 10 point Visual Analog scale **Preference**: satisfaction, comprehensiveness **Communication**: GP counseling frequency and duration, self-confidence	NS patients estimated motivation NS comprehensiveness, satisfaction NS counseling duration, self-confidence Feasibility and acceptability of adding a visual element is “equally high”
**Shahab et al**^27^ **(2007)** Pilot RCT Patients randomized by computer	23 CVD outpatients UK	N=11 **Print of ultrasound image** of their carotid artery alongside a disease-free artery + leaflet linking smoking and CVD	N=12 Routine verbal feedback	Immediately after and at 4 weeks	**Behavior** Intention to stop smoking (7 point Likert scale) Perceived susceptibility Perceived seriousness Perceived response efficacy from smoking cessation Perceived self-efficacy Smoking cessation Qualitative – interviews with patients	All outcomes NS except Perceived susceptibility Mean difference high perceived susceptibility =8.04 (CI: 5.58–10.50) Interviews: Only patients in the intervention group reported the visit made them think seriously about giving up smoking High self-efficacy may be necessary to translate higher risk perception into intention to change behavior
**Saver et al**^28^ **(2014)** Before and after study	English/Spanish speaking adults with T2D and at least 1 CVD risk factor Two general practices in 1 US city	N=56 patients **Verbal** + **Pictorial risk message** First 38 patients randomized to receive bar chart/crowd chart; final 18 patients receive bar chart/crowd chart sequentially	N/A	N/A	**Risk perception** Change in ranking using 6 cards of health risks including mortality **Qualitative data** on reasons for changing/not changing, motivations for change, incongruence in perceptions	NS change in risk ranking Although 80% felt some/all of the data applied to them personally, <40% felt it motivate changes; 75% report “their own body experiences” as their motivator. 20% report a “warning shot” event or an instance where the provider urges, as prompting change Personalized risk estimates have limited salience
**Ahmed et al**^29^ **(2011)** RCT protocol Block randomization	18–69 years Asthma patients from tertiary care pulmonary clinics, Canada N=80	**Web-based self-management** system with asthma status presented as Red (be careful), Amber (needs improvement), Green (keep up the good work) + links to online educational resources tailored to patients’ gaps in knowledge and clinical information	Usual care	3, 6, and 9 months	**Behavior** Chronic disease self-efficacy Medication adherence Health care use	
**Weyman et al**^30^ **(2013)** Outline of an intervention	T2D patients	**Tailored web-based interactive health communication application** Personalization involves mirroring what the user says, conveying esteem and empathy, building individualized bridges, content matching, and presenting users with information on themselves	N/A	N/A	N/A	

**Abbreviations:** BMI, body mass index; CI, confidence interval; CVD, cardiovascular disease; HRQoL, health-related quality of life; GP, general practitioner; HRA, health risk appraisal; ICB, intention to change behavior; N/A, not applicable; NS, not significant; OR, odds ratio; PID, patient initiation of health-related discussion; PN, practice nurse; RCT, randomized controlled trial; T2D, type 2 diabetes.

**Table 3:** Risk of bias of included RCTs

Risk domain	Dapp et al^20^ (2011)	Harari et al^21^ (2008)	Kreuter and Strecher^22^ (1995)	Zullig et al^23^ (2014)	Welschen et al^24^ (2012)	Hess et al^25^ (2014)	Neuner-Jehle et al^26^ (2013)	Shahab et al^27^ (2007)
**Selection bias**								
Random sequence generation	Low	Low	Unclear	Medium	Low	Low	Unclear	Low
Allocation concealment	Low	Low	Unclear	Medium	Low	Low	Unclear	Low
**Performance bias**								
Blinding of participant and personnel	High	High	High	High	High	High	High	High
**Detection bias**								
Blinding of outcome assessment	High	High	Unclear	Unclear	High	High	High	High
**Attrition bias**								
Incomplete outcome data	Low	Low	Low	Unclear	Low	Low	Low	Low
**Reporting bias**								
Selective reporting	Low	Low	High	Low	Unclear	Low	Low	Low
**Other bias**								
Bias other than those above	N/A	N/A	N/A	N/A	N/A	N/A	N/A	N/A

**Abbreviations:** N/A, not applicable; RCT, randomized controlled trial.
